# Editorial: Understanding the influences of sex and gender differences in mental disorders

**DOI:** 10.3389/fpsyt.2022.984195

**Published:** 2022-08-08

**Authors:** Dorte M. Christiansen, Margaret M. McCarthy, Mary V. Seeman

**Affiliations:** ^1^Department of Psychology, Faculty of Health Sciences, University of Southern Denmark, Odense, Denmark; ^2^Department of Pharmacology, School of Medicine, University of Maryland, Baltimore, MD, United States; ^3^Department of Psychiatry, Faculty of Medicine, University of Toronto, Toronto, ON, Canada

**Keywords:** sex, gender, mental health, diathesis-stress model, editorial

From the time of conception and until old age, sex and gender shape humans in a multitude of ways, leading to marked gender differences across most mental disorders. Women have higher rates of internalizing disorders (e.g., depression, anxiety, eating disorders, OCD, borderline personality disorder) and a higher risk of suicide attempts, whereas men have a higher prevalence of externalizing disorders (e.g., substance use disorders, antisocial personality disorder, oppositional defiant disorder) and a higher rate of completed suicide, as well as higher rates of neurodevelopmental disorders (e.g., ADHD, autism, learning disorders). Even in disorders such as schizophrenia, where prevalence rates are similar, sex/gender differences exist in age of onset and severity of symptoms.

The aim of this collection of articles is to go beyond reporting sex/gender differences, to attempt, instead, to understand the many pathways by which sex and gender influence mental disorders. This collection includes seven articles, six original studies and one hypothesis and theory paper by the authors of this editorial, which proposes a diathesis-stress model of sex and gender differences across different disorders, with PTSD, ASD, and eating disorders used to illustrate the model. We will now position the results of the remaining six papers within the framework of the model.

Wang and Jiao's results show that the bidirectional associations between depression in adolescence and parental care are moderated by gender. Whereas, parental care at age 11 is a protective factor against depressive symptoms at age 13 in both boys and girls, depressive symptoms at age 11 predict reduced quality of parental care at age 13 only in boys. In contrast, the paper by Voges et al. suggests that gender stereotypes that result in a drive for thinness in girls and women, may be responsible for young women applying self-deprecating double standards to their bodies, judging body images paired with their own faces, even ideal body images, more harshly than when the same bodies are paired with anonymous faces. Similar pairings of face and body did not produce the same self-critical effect in men. Both of these papers accord with evidence reviewed by Christiansen et al. that early socialization processes encourage physical exploration and toughness in boys in contrast to emotional communication, tenderness, and focus on appearance in girls. Whereas, the findings by Voges et al. suggest that harsh judgement of their own healthy bodies and engagement in negative body talk serves as a mediator between being socialized to place high value on physical appearance and the development of eating disorders in girls, the findings by Wang and Jiao show how gender-incongruent behavior in boys (depression) elicits a probably unconscious reduction in parental care. Similarly, Gomez-Baya et al. suggest that their findings, that gender differences in anxiety among university students are mediated by developmental assets, can be explained by gender socialization. Anxiety in girls, their findings suggest, is a product of an impaired sense of positive identity because, characteristically, girls are encouraged to be interdependent, connected to others, and sensitive to the needs of others while boys develop a more positive identity, having generally been encouraged to independently achieve, accomplish, and attain.

Returning to the diathesis-stress model proposed by Christiansen et al., two studies in this collection examined the effects of a specific stressor—the COVID-19 pandemic. Penengo et al. studied 325 pregnant women during the pandemic in Italy and found high levels of psychiatric symptoms, such as anxiety (43%), depression (10%), and OCD (13%). While pandemic stress did not cause symptom severity above that attributed to pregnancy alone, the authors argue that the pandemic probably increased pregnancy stress by decreasing access to needed healthcare. While pregnancy as a biological stressor is certainly unique to women in terms of extensive hormonal alterations, it may serve as a major psychosocial (gender-specific role expectations) stressor to both men and women. Furthermore, while pregnancy may contribute to other stressors (e.g., relationship, financial), many major external stressors such as the pandemic, or domestic abuse, and even minor stressors such as routine domestic chores and childcare, affect women most strongly during vulnerable periods. Examining the impact of the COVID-19 pandemic on healthcare workers in Spain, López-Atanes et al. found that, in addition to female healthcare workers being over-represented in healthcare jobs of low pay and low status, they were also most at risk for infection. Moreover, women healthcare workers took on additional burdens at home during the pandemic (e.g., helping children with remote learning, caring for vulnerable elderly parents), all of which resulted in sex/gender differential stress leading to a decline in mental health for women.

Mirkovic et al. examined gender differences in suicidal behavior among 320 adolescents and found that girls had attempted suicide more times and started at an earlier age compared to boys. However, and this is the classical gender/suicide paradox, boys used more aggressive and, therefore, more lethal means. That, and the fact that boys/men do not readily seek help when distressed, as stated by López-Atanes et al., contributes to the higher suicide rate in men. Specific psychiatric diagnoses whose rates or severity differ in women (borderline personality disorder) and men (substance use and schizophrenia) probably also contribute to the lethality of suicide attempts.

In support of the Christiansen et al. diathesis-stress model, several of the above studies illustrate the role of pre-existing vulnerability, such as prior mental health diagnoses and stressors, influencing response to new stressors (e.g., COVID-19 or pregnancy), and the role of post-stress coping (spirituality, avoidance, support seeking) in, together, determining the state of mental wellbeing. All these factors, because of biology and/or social learning, bear different weights in men and women and, thus, result in differing expression of mental illness.

The studies included in this Research Topic highlight the importance of paying attention to sex and gender in mental health. Understanding the influences of sex and gender will help not only in designing more effective interventions for men and women (as suggested by both Gomez-Baya et al. and Mirkovic et al.), but also in better addressing variations within each sex/gender.

## Author contributions

All authors listed have made a substantial, direct, and intellectual contribution to the work and approved it for publication.

## Conflict of interest

The authors declare that the research was conducted in the absence of any commercial or financial relationships that could be construed as a potential conflict of interest.

## Publisher's note

All claims expressed in this article are solely those of the authors and do not necessarily represent those of their affiliated organizations, or those of the publisher, the editors and the reviewers. Any product that may be evaluated in this article, or claim that may be made by its manufacturer, is not guaranteed or endorsed by the publisher.

